# Triimidazolium tris­(pyridine-2,6-di­carboxyl­ato)dysprosate(III) trihydrate

**DOI:** 10.1107/S1600536810036421

**Published:** 2010-09-25

**Authors:** Lingzong Meng, Chaowen Duan

**Affiliations:** a School of Chemistry & Environmental Resources, Linyi Normal University, Linyi 276005, People’s Republic of China

## Abstract

The structure of the title compound, (C_3_N_2_H_5_)_3_[Dy(C_7_H_3_NO_4_)_3_]·3H_2_O, contains a mononuclear Dy^III^ complex with the rare earth metal cation in a distorted tricapped trigonal–prismatic environment. The Dy^III^ ion is in each case *O*,*N*,*O*′-chelated by three tridentate pyridine-2,6-dicarboxyl­ate anions. Three protonated imidazole mol­ecules act as counter-cations and three lattice water mol­ecules are also present. Numerous N—H⋯O and O—H⋯O hydrogen bonding inter­actions, some of which are bifurcated, help to stabilize the packing of the structure.

## Related literature

For background to pyridine-2,6-dicarb­oxy­lic acid (H_2_pda) and structures of metal complexes with (pda^2−^) ligands, see: Ghosh & Bharadwaj (2005 ▶); Huang *et al.* (2008 ▶); Kjell *et al.* (1993 ▶); Song *et al.* (2005 ▶); Wu *et al.* (2008 ▶); Yue *et al.* (2005 ▶); Zhao *et al.* (2005 ▶, 2007 ▶).
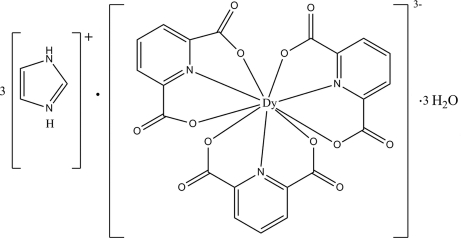


## Experimental

### 

#### Crystal data

(C_3_H_5_N_2_)_3_[Dy(C_7_H_3_NO_4_)_3_]·3H_2_O
*M* *_r_* = 919.13
Triclinic, 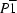

*a* = 10.939 (2) Å
*b* = 12.099 (2) Å
*c* = 14.070 (3) Å
α = 88.57 (3)°
β = 85.64 (3)°
γ = 67.28 (3)°
*V* = 1712.7 (6) Å^3^
*Z* = 2
Mo *K*α radiation
μ = 2.27 mm^−1^
*T* = 296 K
0.35 × 0.25 × 0.25 mm

#### Data collection

Bruker APEXII CCD diffractometer
Absorption correction: multi-scan (*SADABS*; Bruker, 2001 ▶) *T*_min_ = 0.504, *T*_max_ = 0.601
27464 measured reflections
7447 independent reflections
7223 reflections with *I* > 2σ(*I*)
*R*_int_ = 0.022

#### Refinement

*R*[*F* ^2^ > 2σ(*F* ^2^)] = 0.022
*wR*(*F* ^2^) = 0.060
*S* = 1.10
7447 reflections
505 parameters
3 restraints
H atoms treated by a mixture of independent and constrained refinement
Δρ_max_ = 0.69 e Å^−3^
Δρ_min_ = −0.77 e Å^−3^

### 

Data collection: *APEX2* (Bruker, 2008 ▶); cell refinement: *SAINT* (Bruker, 2008 ▶); data reduction: *SAINT*; program(s) used to solve structure: *SHELXS97* (Sheldrick, 2008 ▶); program(s) used to refine structure: *SHELXL97* (Sheldrick, 2008 ▶); molecular graphics: *SHELXTL* (Sheldrick, 2008 ▶); software used to prepare material for publication: *SHELXTL*.

## Supplementary Material

Crystal structure: contains datablocks I, global. DOI: 10.1107/S1600536810036421/wm2398sup1.cif

Structure factors: contains datablocks I. DOI: 10.1107/S1600536810036421/wm2398Isup2.hkl

Additional supplementary materials:  crystallographic information; 3D view; checkCIF report

## Figures and Tables

**Table 1:** Selected bond lengths (Å)

Dy2—O5	2.3745 (19)
Dy2—O1	2.4032 (17)
Dy2—O7	2.4072 (18)
Dy2—O3	2.4167 (19)
Dy2—O11	2.420 (2)
Dy2—O9	2.426 (2)
Dy2—N2	2.482 (2)
Dy2—N1	2.492 (2)
Dy2—N3	2.506 (2)

**Table 2:** Hydrogen-bond geometry (Å, °)

*D*—H⋯*A*	*D*—H	H⋯*A*	*D*⋯*A*	*D*—H⋯*A*
N4—H4*A*⋯O14^i^	0.94 (2)	2.50 (3)	3.286 (4)	141 (4)
N5—H5⋯O12^i^	0.86	2.27	3.121 (5)	171
N8—H8*A*⋯O10^ii^	0.96 (2)	2.29 (2)	3.241 (4)	172 (4)
N7—H7*A*⋯O10^iii^	0.87 (2)	1.82 (2)	2.695 (4)	177 (4)
N7—H7*A*⋯O9^iii^	0.87 (2)	2.60 (3)	3.127 (3)	120 (3)
N6—H6⋯O14^iv^	0.86	1.89	2.731 (4)	167
O13—H2*W*⋯O15^iv^	0.85	2.02	2.828 (4)	159
O13—H1*W*⋯O11	0.85	2.05	2.896 (3)	174
O13—H1*W*⋯O12	0.85	2.61	3.192 (4)	127
O14—H3*W*⋯O4	0.85	1.91	2.756 (3)	178
O14—H4*W*⋯O6^v^	0.85	2.06	2.842 (3)	153
O15—H5*W*⋯O2^vi^	0.85	2.22	3.061 (4)	169
O15—H6*W*⋯O4	0.85	2.00	2.845 (4)	172

Symmetry codes: (i) 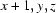; (ii) 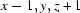; (iii) 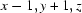; (iv) 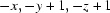; (v) 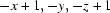; (vi) 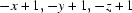.

**Fig. 1.**
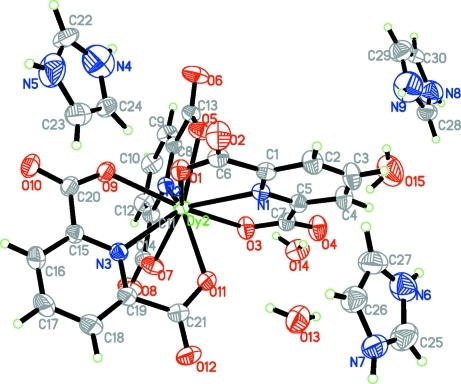
The asymmetric unit of the title compound, showing displacement ellipsoids at the 50% probability level.

## supplementary crystallographic information

### Comment

In recent years, the interaction of pyridine-2,6-dicarboxylic acid (H_2_pdc) with several metal ions has been extensively studied due to its unique ability to form stable chelates in diverse coordination modes such as bidentate, meridian and bridging (Kjell *et al.*, 1993). A considerable number of metal—pdc complexes have been synthesized and their structures determined over the past decade (Huang *et al.*, 2008; Ghosh *et al.*, 2005; Song *et al.*, 2005; Wu *et al.*, 2008; Yue *et al.*, 2005; Zhao *et al.*, 2005, 2007). Here we present the structure of the title compound (C_3_N_2_H_5_)_3_[Dy(C_7_H_3_NO_4_)_3_]^.^(H_2_O)_3_, which includes pyridinedicarboxylate (pdc^2-^) anions and imidazolium (im) counter cations.

The crystal structure is composed of a mononuclear Dy^III^ complex with the rare earth metal cation in a distorted tricapped trigonal-prismatic environment (Fig. 1, Table 1). The Dy^III^ ion is in each case *O,N,O*-chelated by three tridentate pyridine-2,6-dicarboxylate (pda^2-^) ligands. Three imidazolium molecules act as counter cations. Moreover, three lattice water molecules are present. Numerous N—H···O, O—H···O and O—H···N hydrogen bonding interactions (Table 2), part of which are bifurcated, lead to a three-dimensional assembly of the structural building blocks.

### Experimental

The title compound was synthesized under solvothermal conditions. A mixture of pyridine-2,6-dicarboxylic acid (0.0334 g, 0.2 mmol), Dy(NO_3_)_3_^.^6H_2_O (0.0245 g, 0.06 mmol), imidazole (0.0340 g, 0.5 mmol) and H_2_O / C_2_H_5_OH (v / v = 1: 1, 2.5 ml) was sealed in a 6 ml glass tube and heated to 393 K for 72 h. After cooling to room temperature, colorless block-like crystals were obtained.

### Refinement

H atoms bound to C and N atoms were placed in calculated positions with C—H = 0.93 and N—H = 0.86 Å and refined in riding mode, with *U*_iso_(H) = 1.2 *U*_eq_(N, C). H atoms attached to water molecules were located in Fourier maps and refined with distance constraints of 0.85 Å and *U*_iso_(H) = 1.5 *U*_eq_(O).

### Crystal data

**Table:**

(C_3_H_5_N_2_)_3_[Dy(C_7_H_3_NO_4_)_3_]·3H_2_O	*Z* = 2
*M**_r_* = 919.13	*F*(000) = 918
Triclinic, *P*1	*D*_x_ = 1.782 Mg m^−^^3^
Hall symbol: -P 1	Mo *K*α radiation, λ = 0.71073 Å
*a* = 10.939 (2) Å	Cell parameters from 125 reflections
*b* = 12.099 (2) Å	θ = 7.5–15°
*c* = 14.070 (3) Å	µ = 2.27 mm^−^^1^
α = 88.57 (3)°	*T* = 296 K
β = 85.64 (3)°	Block, colourless
γ = 67.28 (3)°	0.35 × 0.25 × 0.25 mm
*V* = 1712.7 (6) Å^3^	

### Data collection

**Table:**

Bruker APEXII CCD diffractometer	7447 independent reflections
Radiation source: fine-focus sealed tube	7223 reflections with *I* > 2σ(*I*)
graphite	*R*_int_ = 0.022
φ and ω scans	θ_max_ = 27.0°, θ_min_ = 1.8°
Absorption correction: multi-scan (*SADABS*; Bruker, 2001)	*h* = −13→13
*T*_min_ = 0.504, *T*_max_ = 0.601	*k* = −13→15
27464 measured reflections	*l* = −17→17

### Refinement

**Table:**

Refinement on *F*^2^	Primary atom site location: structure-invariant direct methods
Least-squares matrix: full	Secondary atom site location: difference Fourier map
*R*[*F*^2^ > 2σ(*F*^2^)] = 0.022	Hydrogen site location: inferred from neighbouring sites
*wR*(*F*^2^) = 0.060	H atoms treated by a mixture of independent and constrained refinement
*S* = 1.10	*w* = 1/[σ^2^(*F*_o_^2^) + (0.0314*P*)^2^ + 1.7104*P*] where *P* = (*F*_o_^2^ + 2*F*_c_^2^)/3
7447 reflections	(Δ/σ)_max_ = 0.001
505 parameters	Δρ_max_ = 0.69 e Å^−^^3^
3 restraints	Δρ_min_ = −0.77 e Å^−^^3^

### Special details

**Table:**

Geometry. All e.s.d.'s (except the e.s.d. in the dihedral angle between two l.s. planes) are estimated using the full covariance matrix. The cell e.s.d.'s are taken into account individually in the estimation of e.s.d.'s in distances, angles and torsion angles; correlations between e.s.d.'s in cell parameters are only used when they are defined by crystal symmetry. An approximate (isotropic) treatment of cell e.s.d.'s is used for estimating e.s.d.'s involving l.s. planes.
Refinement. Refinement of *F*^2^ against ALL reflections. The weighted *R*-factor *wR* and goodness of fit *S* are based on *F*^2^, conventional *R*-factors *R* are based on *F*, with *F* set to zero for negative *F*^2^. The threshold expression of *F*^2^ > σ(*F*^2^) is used only for calculating *R*-factors(gt) *etc*. and is not relevant to the choice of reflections for refinement. *R*-factors based on *F*^2^ are statistically about twice as large as those based on *F*, and *R*- factors based on ALL data will be even larger.

### Fractional atomic coordinates and isotropic or equivalent isotropic displacement parameters (Å^2^)

**Table:**

	*x*	*y*	*z*	*U*_iso_*/*U*_eq_	
Dy2	0.492045 (10)	0.266222 (9)	0.252168 (7)	0.02219 (4)	
N1	0.38988 (19)	0.43970 (17)	0.36385 (14)	0.0243 (4)	
O11	0.31544 (18)	0.41655 (17)	0.17113 (13)	0.0338 (4)	
N2	0.5350 (2)	0.05867 (18)	0.30655 (14)	0.0257 (4)	
O3	0.29744 (19)	0.27164 (17)	0.34879 (14)	0.0345 (4)	
O9	0.71488 (18)	0.14570 (17)	0.18726 (13)	0.0344 (4)	
O7	0.40575 (19)	0.15338 (17)	0.15780 (13)	0.0348 (4)	
O1	0.60328 (17)	0.40305 (16)	0.25167 (13)	0.0304 (4)	
O5	0.6077 (2)	0.20866 (16)	0.39291 (13)	0.0350 (4)	
N3	0.5446 (2)	0.29826 (18)	0.07972 (14)	0.0265 (4)	
O10	0.8795 (2)	0.1004 (2)	0.07273 (15)	0.0456 (5)	
C6	0.5682 (3)	0.4965 (2)	0.30392 (18)	0.0288 (5)	
C14	0.4159 (3)	0.0481 (2)	0.17445 (18)	0.0298 (5)	
C13	0.6477 (3)	0.1058 (2)	0.42917 (18)	0.0300 (5)	
C5	0.2800 (2)	0.4523 (2)	0.41906 (17)	0.0269 (5)	
C1	0.4427 (2)	0.5216 (2)	0.36810 (17)	0.0268 (5)	
O8	0.3703 (2)	−0.01097 (19)	0.12661 (15)	0.0432 (5)	
C15	0.6663 (2)	0.2410 (2)	0.03876 (18)	0.0290 (5)	
C8	0.6011 (2)	0.0175 (2)	0.38414 (17)	0.0270 (5)	
C21	0.3162 (3)	0.4294 (3)	0.0813 (2)	0.0388 (6)	
O6	0.7192 (2)	0.07182 (19)	0.49629 (16)	0.0475 (5)	
C11	0.5093 (3)	−0.1261 (2)	0.2908 (2)	0.0368 (6)	
H11	0.4774	−0.1736	0.2576	0.044*	
O4	0.1420 (2)	0.34799 (19)	0.46686 (15)	0.0419 (5)	
C12	0.4892 (2)	−0.0110 (2)	0.26079 (18)	0.0286 (5)	
C20	0.7622 (3)	0.1555 (2)	0.10429 (19)	0.0313 (5)	
C7	0.2347 (2)	0.3501 (2)	0.41110 (18)	0.0282 (5)	
O2	0.6264 (2)	0.5655 (2)	0.30446 (17)	0.0466 (5)	
C2	0.3867 (3)	0.6207 (2)	0.4278 (2)	0.0363 (6)	
H2	0.4260	0.6762	0.4306	0.044*	
C9	0.6241 (3)	−0.0961 (2)	0.4192 (2)	0.0349 (6)	
H9	0.6695	−0.1231	0.4739	0.042*	
C4	0.2156 (3)	0.5508 (2)	0.4781 (2)	0.0355 (6)	
H4	0.1369	0.5601	0.5135	0.043*	
C19	0.4500 (3)	0.3706 (2)	0.02714 (18)	0.0305 (5)	
C16	0.6994 (3)	0.2573 (3)	−0.0559 (2)	0.0388 (6)	
H16	0.7858	0.2185	−0.0824	0.047*	
C17	0.6007 (3)	0.3327 (3)	−0.1099 (2)	0.0445 (7)	
H17	0.6199	0.3450	−0.1739	0.053*	
C18	0.4737 (3)	0.3896 (3)	−0.0690 (2)	0.0413 (6)	
H18	0.4057	0.4393	−0.1047	0.050*	
C10	0.5776 (3)	−0.1689 (2)	0.3708 (2)	0.0391 (6)	
H10	0.5924	−0.2460	0.3922	0.047*	
O12	0.2182 (2)	0.4833 (3)	0.03738 (18)	0.0843 (11)	
C3	0.2725 (3)	0.6355 (3)	0.4827 (2)	0.0418 (7)	
H3	0.2332	0.7017	0.5229	0.050*	
C30	0.2356 (3)	0.1317 (3)	0.9875 (2)	0.0349 (6)	
H30	0.2716	0.0773	1.0356	0.042*	
C29	0.3007 (4)	0.1858 (4)	0.9312 (3)	0.0565 (9)	
H29	0.3893	0.1754	0.9332	0.068*	
N9	0.2143 (4)	0.2573 (4)	0.8719 (3)	0.0879 (12)	
H9A	0.2305	0.3022	0.8285	0.105*	
C28	0.0964 (3)	0.2459 (3)	0.8928 (2)	0.0409 (6)	
H28	0.0184	0.2856	0.8628	0.049*	
N8	0.1118 (4)	0.1690 (4)	0.9629 (3)	0.0694 (9)	
H8A	0.044 (4)	0.144 (4)	0.990 (3)	0.083*	
N7	0.0067 (3)	0.9748 (3)	0.22123 (19)	0.0460 (6)	
H7A	−0.032 (3)	1.016 (3)	0.172 (2)	0.055*	
C25	−0.0575 (3)	0.9456 (4)	0.2928 (3)	0.0577 (9)	
H25	−0.1468	0.9570	0.2959	0.069*	
C26	0.1345 (3)	0.9451 (3)	0.2428 (2)	0.0461 (7)	
H26	0.2019	0.9561	0.2043	0.055*	
N6	0.0242 (3)	0.8977 (3)	0.3600 (2)	0.0556 (7)	
H6	0.0043	0.8714	0.4138	0.067*	
C27	0.1448 (4)	0.8969 (3)	0.3299 (3)	0.0520 (8)	
H27	0.2209	0.8682	0.3635	0.062*	
C23	0.9146 (3)	0.3939 (4)	0.1412 (2)	0.0529 (8)	
H23	0.8654	0.4415	0.0937	0.063*	
C24	0.8704 (3)	0.3407 (3)	0.2103 (2)	0.0363 (6)	
H24	0.7834	0.3455	0.2205	0.044*	
C22	1.0765 (3)	0.2936 (3)	0.2290 (3)	0.0520 (8)	
H22	1.1600	0.2603	0.2529	0.062*	
N5	1.0434 (3)	0.3669 (4)	0.1519 (3)	0.0713 (10)	
H5	1.0957	0.3912	0.1167	0.086*	
O13	0.0930 (3)	0.6349 (2)	0.22816 (19)	0.0625 (7)	
H2W	0.0469	0.6143	0.2710	0.094*	
H1W	0.1609	0.5740	0.2092	0.094*	
O14	0.0736 (2)	0.1513 (2)	0.46679 (16)	0.0482 (5)	
H3W	0.0936	0.2127	0.4652	0.072*	
H4W	0.1461	0.0904	0.4598	0.072*	
O15	0.1116 (3)	0.4098 (3)	0.6635 (2)	0.0794 (9)	
H5W	0.1782	0.4267	0.6729	0.119*	
H6W	0.1159	0.3982	0.6038	0.119*	
N4	0.9700 (4)	0.2789 (3)	0.2631 (3)	0.0758 (10)	
H4A	0.966 (5)	0.231 (4)	0.316 (3)	0.091*	

### Atomic displacement parameters (Å^2^)

**Table:**

	*U*^11^	*U*^22^	*U*^33^	*U*^12^	*U*^13^	*U*^23^
Dy2	0.02178 (6)	0.02328 (6)	0.02335 (7)	−0.01066 (4)	−0.00168 (4)	−0.00032 (4)
N1	0.0258 (10)	0.0247 (9)	0.0244 (9)	−0.0116 (8)	−0.0031 (8)	0.0020 (7)
O11	0.0279 (9)	0.0380 (10)	0.0295 (9)	−0.0059 (7)	−0.0021 (7)	0.0010 (7)
N2	0.0259 (10)	0.0265 (9)	0.0266 (10)	−0.0127 (8)	−0.0001 (8)	−0.0016 (8)
O3	0.0353 (10)	0.0355 (9)	0.0383 (10)	−0.0213 (8)	0.0089 (8)	−0.0095 (8)
O9	0.0272 (9)	0.0370 (10)	0.0327 (10)	−0.0062 (7)	0.0007 (7)	0.0046 (8)
O7	0.0442 (11)	0.0354 (9)	0.0324 (9)	−0.0221 (8)	−0.0110 (8)	0.0018 (7)
O1	0.0286 (9)	0.0306 (9)	0.0349 (9)	−0.0156 (7)	0.0033 (7)	−0.0038 (7)
O5	0.0469 (11)	0.0284 (9)	0.0335 (10)	−0.0166 (8)	−0.0143 (8)	0.0015 (7)
N3	0.0265 (10)	0.0279 (10)	0.0261 (10)	−0.0115 (8)	−0.0020 (8)	−0.0001 (8)
O10	0.0290 (10)	0.0535 (13)	0.0418 (11)	−0.0038 (9)	0.0053 (8)	0.0010 (9)
C6	0.0294 (12)	0.0286 (11)	0.0321 (13)	−0.0151 (10)	−0.0045 (10)	0.0031 (9)
C14	0.0282 (12)	0.0353 (13)	0.0298 (12)	−0.0168 (10)	0.0000 (10)	−0.0045 (10)
C13	0.0334 (13)	0.0297 (12)	0.0282 (12)	−0.0133 (10)	−0.0045 (10)	−0.0008 (9)
C5	0.0279 (12)	0.0271 (11)	0.0256 (11)	−0.0108 (9)	−0.0008 (9)	0.0015 (9)
C1	0.0293 (12)	0.0262 (11)	0.0277 (12)	−0.0133 (9)	−0.0045 (9)	0.0019 (9)
O8	0.0535 (12)	0.0449 (11)	0.0446 (11)	−0.0312 (10)	−0.0158 (10)	−0.0016 (9)
C15	0.0276 (12)	0.0324 (12)	0.0278 (12)	−0.0128 (10)	−0.0004 (9)	−0.0022 (9)
C8	0.0271 (12)	0.0279 (11)	0.0264 (12)	−0.0111 (9)	−0.0002 (9)	−0.0005 (9)
C21	0.0306 (13)	0.0470 (16)	0.0327 (14)	−0.0074 (12)	−0.0071 (11)	0.0016 (12)
O6	0.0633 (14)	0.0397 (11)	0.0435 (12)	−0.0200 (10)	−0.0295 (11)	0.0068 (9)
C11	0.0399 (15)	0.0316 (13)	0.0454 (15)	−0.0207 (11)	−0.0034 (12)	−0.0024 (11)
O4	0.0411 (11)	0.0447 (11)	0.0454 (12)	−0.0255 (9)	0.0149 (9)	−0.0085 (9)
C12	0.0286 (12)	0.0300 (12)	0.0312 (12)	−0.0158 (10)	−0.0008 (10)	−0.0025 (10)
C20	0.0256 (12)	0.0313 (12)	0.0350 (14)	−0.0090 (10)	−0.0001 (10)	−0.0028 (10)
C7	0.0269 (12)	0.0307 (12)	0.0290 (12)	−0.0136 (10)	−0.0002 (9)	0.0015 (9)
O2	0.0484 (12)	0.0445 (11)	0.0597 (14)	−0.0336 (10)	0.0092 (10)	−0.0107 (10)
C2	0.0434 (15)	0.0301 (12)	0.0396 (15)	−0.0191 (11)	0.0000 (12)	−0.0049 (11)
C9	0.0339 (13)	0.0321 (13)	0.0399 (14)	−0.0138 (11)	−0.0055 (11)	0.0069 (11)
C4	0.0378 (14)	0.0338 (13)	0.0348 (14)	−0.0154 (11)	0.0080 (11)	−0.0058 (11)
C19	0.0314 (13)	0.0335 (12)	0.0260 (12)	−0.0118 (10)	−0.0035 (10)	0.0020 (10)
C16	0.0351 (14)	0.0504 (16)	0.0304 (13)	−0.0170 (12)	0.0055 (11)	−0.0030 (12)
C17	0.0501 (18)	0.0593 (19)	0.0244 (13)	−0.0221 (15)	0.0007 (12)	0.0048 (12)
C18	0.0438 (16)	0.0486 (16)	0.0296 (14)	−0.0155 (13)	−0.0068 (12)	0.0077 (12)
C10	0.0413 (15)	0.0291 (13)	0.0514 (17)	−0.0186 (11)	−0.0044 (13)	0.0082 (12)
O12	0.0377 (13)	0.131 (3)	0.0416 (14)	0.0155 (15)	−0.0127 (11)	0.0054 (15)
C3	0.0505 (17)	0.0311 (13)	0.0430 (16)	−0.0161 (12)	0.0083 (13)	−0.0122 (12)
C30	0.0391 (14)	0.0399 (14)	0.0301 (13)	−0.0187 (12)	−0.0093 (11)	−0.0013 (11)
C29	0.0447 (18)	0.069 (2)	0.057 (2)	−0.0227 (17)	−0.0042 (16)	0.0032 (17)
N9	0.097 (3)	0.088 (3)	0.072 (2)	−0.031 (2)	0.000 (2)	0.019 (2)
C28	0.0372 (15)	0.0498 (16)	0.0341 (14)	−0.0145 (13)	−0.0077 (12)	0.0051 (12)
N8	0.069 (2)	0.079 (2)	0.068 (2)	−0.0367 (19)	−0.0072 (17)	−0.0045 (18)
N7	0.0380 (13)	0.0514 (15)	0.0421 (14)	−0.0099 (11)	−0.0052 (11)	0.0048 (12)
C25	0.0390 (17)	0.072 (2)	0.056 (2)	−0.0163 (16)	0.0029 (15)	0.0062 (18)
C26	0.0381 (15)	0.0491 (17)	0.0527 (18)	−0.0185 (13)	−0.0025 (13)	−0.0035 (14)
N6	0.0671 (19)	0.0552 (17)	0.0408 (15)	−0.0205 (15)	0.0000 (13)	0.0057 (12)
C27	0.0521 (19)	0.0497 (18)	0.056 (2)	−0.0181 (15)	−0.0202 (16)	−0.0003 (15)
C23	0.0414 (17)	0.073 (2)	0.0451 (18)	−0.0223 (16)	−0.0081 (14)	0.0018 (16)
C24	0.0236 (12)	0.0502 (16)	0.0407 (15)	−0.0206 (11)	0.0011 (10)	−0.0094 (12)
C22	0.0242 (14)	0.061 (2)	0.064 (2)	−0.0068 (13)	−0.0130 (14)	−0.0145 (17)
N5	0.0547 (19)	0.094 (3)	0.079 (2)	−0.0465 (19)	0.0196 (17)	−0.026 (2)
O13	0.0524 (14)	0.0571 (15)	0.0597 (15)	−0.0014 (12)	−0.0012 (12)	−0.0022 (12)
O14	0.0477 (12)	0.0453 (12)	0.0567 (14)	−0.0235 (10)	−0.0070 (10)	0.0091 (10)
O15	0.090 (2)	0.102 (2)	0.0473 (15)	−0.0424 (19)	0.0164 (14)	−0.0014 (15)
N4	0.093 (3)	0.058 (2)	0.071 (2)	−0.023 (2)	−0.009 (2)	0.0016 (17)

### Geometric parameters (Å, °)

**Table:**

Dy2—O5	2.3745 (19)	C4—H4	0.9300
Dy2—O1	2.4032 (17)	C19—C18	1.388 (4)
Dy2—O7	2.4072 (18)	C16—C17	1.381 (4)
Dy2—O3	2.4167 (19)	C16—H16	0.9300
Dy2—O11	2.420 (2)	C17—C18	1.376 (4)
Dy2—O9	2.426 (2)	C17—H17	0.9300
Dy2—N2	2.482 (2)	C18—H18	0.9300
Dy2—N1	2.492 (2)	C10—H10	0.9300
Dy2—N3	2.506 (2)	C3—H3	0.9300
N1—C1	1.331 (3)	C30—N8	1.322 (4)
N1—C5	1.339 (3)	C30—C29	1.345 (5)
O11—C21	1.270 (3)	C30—H30	0.9300
N2—C8	1.333 (3)	C29—N9	1.339 (5)
N2—C12	1.334 (3)	C29—H29	0.9300
O3—C7	1.260 (3)	N9—C28	1.359 (5)
O9—C20	1.262 (3)	N9—H9A	0.8600
O7—C14	1.253 (3)	C28—N8	1.313 (5)
O1—C6	1.277 (3)	C28—H28	0.9300
O5—C13	1.258 (3)	N8—H8A	0.960 (19)
N3—C19	1.330 (3)	N7—C25	1.304 (4)
N3—C15	1.333 (3)	N7—C26	1.359 (4)
O10—C20	1.246 (3)	N7—H7A	0.873 (18)
C6—O2	1.230 (3)	C25—N6	1.315 (5)
C6—C1	1.515 (4)	C25—H25	0.9300
C14—O8	1.250 (3)	C26—C27	1.338 (5)
C14—C12	1.514 (4)	C26—H26	0.9300
C13—O6	1.232 (3)	N6—C27	1.351 (5)
C13—C8	1.518 (3)	N6—H6	0.8600
C5—C4	1.385 (4)	C27—H27	0.9300
C5—C7	1.510 (3)	C23—C24	1.317 (5)
C1—C2	1.387 (4)	C23—N5	1.337 (5)
C15—C16	1.383 (4)	C23—H23	0.9300
C15—C20	1.511 (4)	C24—N4	1.328 (5)
C8—C9	1.384 (3)	C24—H24	0.9300
C21—O12	1.223 (4)	C22—N4	1.301 (5)
C21—C19	1.511 (4)	C22—N5	1.362 (5)
C11—C10	1.375 (4)	C22—H22	0.9300
C11—C12	1.383 (4)	N5—H5	0.8600
C11—H11	0.9300	O13—H2W	0.8499
O4—C7	1.241 (3)	O13—H1W	0.8500
C2—C3	1.369 (4)	O14—H3W	0.8501
C2—H2	0.9300	O14—H4W	0.8500
C9—C10	1.388 (4)	O15—H5W	0.8500
C9—H9	0.9300	O15—H6W	0.8501
C4—C3	1.395 (4)	N4—H4A	0.937 (19)
			
O5—Dy2—O1	78.70 (7)	O10—C20—O9	125.6 (3)
O5—Dy2—O7	129.04 (6)	O10—C20—C15	118.3 (2)
O1—Dy2—O7	146.45 (6)	O9—C20—C15	116.1 (2)
O5—Dy2—O3	86.53 (7)	O4—C7—O3	125.3 (2)
O1—Dy2—O3	128.79 (6)	O4—C7—C5	118.4 (2)
O7—Dy2—O3	77.27 (7)	O3—C7—C5	116.3 (2)
O5—Dy2—O11	147.58 (7)	C3—C2—C1	119.0 (2)
O1—Dy2—O11	88.66 (7)	C3—C2—H2	120.5
O7—Dy2—O11	75.43 (7)	C1—C2—H2	120.5
O3—Dy2—O11	78.34 (7)	C8—C9—C10	118.3 (3)
O5—Dy2—O9	78.53 (7)	C8—C9—H9	120.8
O1—Dy2—O9	77.03 (7)	C10—C9—H9	120.8
O7—Dy2—O9	89.53 (7)	C5—C4—C3	117.9 (3)
O3—Dy2—O9	147.00 (7)	C5—C4—H4	121.1
O11—Dy2—O9	127.78 (6)	C3—C4—H4	121.1
O5—Dy2—N2	64.77 (7)	N3—C19—C18	122.3 (3)
O1—Dy2—N2	137.08 (6)	N3—C19—C21	114.1 (2)
O7—Dy2—N2	64.30 (7)	C18—C19—C21	123.6 (2)
O3—Dy2—N2	72.61 (7)	C17—C16—C15	118.2 (3)
O11—Dy2—N2	134.24 (7)	C17—C16—H16	120.9
O9—Dy2—N2	74.41 (7)	C15—C16—H16	120.9
O5—Dy2—N1	74.09 (7)	C18—C17—C16	119.8 (3)
O1—Dy2—N1	64.41 (6)	C18—C17—H17	120.1
O7—Dy2—N1	134.39 (7)	C16—C17—H17	120.1
O3—Dy2—N1	64.39 (7)	C17—C18—C19	118.2 (3)
O11—Dy2—N1	73.51 (7)	C17—C18—H18	120.9
O9—Dy2—N1	136.03 (7)	C19—C18—H18	120.9
N2—Dy2—N1	121.12 (7)	C11—C10—C9	119.5 (2)
O5—Dy2—N3	137.66 (7)	C11—C10—H10	120.2
O1—Dy2—N3	74.77 (7)	C9—C10—H10	120.2
O7—Dy2—N3	71.72 (7)	C2—C3—C4	119.6 (3)
O3—Dy2—N3	135.76 (7)	C2—C3—H3	120.2
O11—Dy2—N3	63.97 (7)	C4—C3—H3	120.2
O9—Dy2—N3	63.82 (7)	N8—C30—C29	108.5 (3)
N2—Dy2—N3	118.21 (7)	N8—C30—H30	125.7
N1—Dy2—N3	120.61 (7)	C29—C30—H30	125.7
C1—N1—C5	119.4 (2)	N9—C29—C30	107.2 (3)
C1—N1—Dy2	120.34 (16)	N9—C29—H29	126.4
C5—N1—Dy2	120.26 (15)	C30—C29—H29	126.4
C21—O11—Dy2	124.08 (17)	C29—N9—C28	107.4 (3)
C8—N2—C12	119.5 (2)	C29—N9—H9A	126.3
C8—N2—Dy2	119.67 (16)	C28—N9—H9A	126.3
C12—N2—Dy2	120.78 (16)	N8—C28—N9	108.0 (3)
C7—O3—Dy2	124.83 (16)	N8—C28—H28	126.0
C20—O9—Dy2	125.16 (16)	N9—C28—H28	126.0
C14—O7—Dy2	125.14 (16)	C28—N8—C30	108.8 (3)
C6—O1—Dy2	125.57 (16)	C28—N8—H8A	124 (3)
C13—O5—Dy2	125.83 (16)	C30—N8—H8A	127 (3)
C19—N3—C15	119.0 (2)	C25—N7—C26	108.6 (3)
C19—N3—Dy2	120.24 (17)	C25—N7—H7A	123 (3)
C15—N3—Dy2	120.71 (16)	C26—N7—H7A	128 (3)
O2—C6—O1	125.4 (2)	N7—C25—N6	109.0 (3)
O2—C6—C1	119.6 (2)	N7—C25—H25	125.5
O1—C6—C1	115.0 (2)	N6—C25—H25	125.5
O8—C14—O7	125.7 (2)	C27—C26—N7	106.8 (3)
O8—C14—C12	118.0 (2)	C27—C26—H26	126.6
O7—C14—C12	116.3 (2)	N7—C26—H26	126.6
O6—C13—O5	126.3 (2)	C25—N6—C27	108.3 (3)
O6—C13—C8	118.5 (2)	C25—N6—H6	125.8
O5—C13—C8	115.2 (2)	C27—N6—H6	125.8
N1—C5—C4	122.2 (2)	C26—C27—N6	107.3 (3)
N1—C5—C7	114.0 (2)	C26—C27—H27	126.4
C4—C5—C7	123.8 (2)	N6—C27—H27	126.4
N1—C1—C2	121.8 (2)	C24—C23—N5	107.4 (3)
N1—C1—C6	114.6 (2)	C24—C23—H23	126.3
C2—C1—C6	123.5 (2)	N5—C23—H23	126.3
N3—C15—C16	122.4 (2)	C23—C24—N4	108.9 (3)
N3—C15—C20	113.9 (2)	C23—C24—H24	125.5
C16—C15—C20	123.7 (2)	N4—C24—H24	125.5
N2—C8—C9	122.0 (2)	N4—C22—N5	107.5 (3)
N2—C8—C13	114.1 (2)	N4—C22—H22	126.3
C9—C8—C13	123.9 (2)	N5—C22—H22	126.3
O12—C21—O11	125.0 (3)	C23—N5—C22	107.4 (3)
O12—C21—C19	119.4 (3)	C23—N5—H5	126.3
O11—C21—C19	115.6 (2)	C22—N5—H5	126.3
C10—C11—C12	118.7 (2)	H2W—O13—H1W	109.8
C10—C11—H11	120.6	H3W—O14—H4W	107.0
C12—C11—H11	120.6	H5W—O15—H6W	105.4
N2—C12—C11	121.9 (2)	C22—N4—C24	108.8 (3)
N2—C12—C14	113.5 (2)	C22—N4—H4A	125 (3)
C11—C12—C14	124.6 (2)	C24—N4—H4A	126 (3)
			
O5—Dy2—N1—C1	85.81 (18)	N1—Dy2—N3—C15	126.34 (18)
O1—Dy2—N1—C1	0.95 (16)	Dy2—O1—C6—O2	178.9 (2)
O7—Dy2—N1—C1	−144.58 (16)	Dy2—O1—C6—C1	−2.1 (3)
O3—Dy2—N1—C1	179.64 (19)	Dy2—O7—C14—O8	179.2 (2)
O11—Dy2—N1—C1	−95.64 (18)	Dy2—O7—C14—C12	−0.3 (3)
O9—Dy2—N1—C1	32.2 (2)	Dy2—O5—C13—O6	172.1 (2)
N2—Dy2—N1—C1	132.20 (17)	Dy2—O5—C13—C8	−8.0 (3)
N3—Dy2—N1—C1	−50.61 (19)	C1—N1—C5—C4	2.7 (4)
O5—Dy2—N1—C5	−95.26 (18)	Dy2—N1—C5—C4	−176.21 (19)
O1—Dy2—N1—C5	179.88 (19)	C1—N1—C5—C7	−177.0 (2)
O7—Dy2—N1—C5	34.4 (2)	Dy2—N1—C5—C7	4.1 (3)
O3—Dy2—N1—C5	−1.43 (16)	C5—N1—C1—C2	−0.4 (4)
O11—Dy2—N1—C5	83.29 (17)	Dy2—N1—C1—C2	178.49 (19)
O9—Dy2—N1—C5	−148.86 (16)	C5—N1—C1—C6	178.8 (2)
N2—Dy2—N1—C5	−48.87 (19)	Dy2—N1—C1—C6	−2.2 (3)
N3—Dy2—N1—C5	128.32 (17)	O2—C6—C1—N1	−178.2 (2)
O5—Dy2—O11—C21	153.0 (2)	O1—C6—C1—N1	2.7 (3)
O1—Dy2—O11—C21	86.7 (2)	O2—C6—C1—C2	1.1 (4)
O7—Dy2—O11—C21	−63.5 (2)	O1—C6—C1—C2	−178.0 (2)
O3—Dy2—O11—C21	−143.2 (2)	C19—N3—C15—C16	2.4 (4)
O9—Dy2—O11—C21	14.3 (3)	Dy2—N3—C15—C16	−179.3 (2)
N2—Dy2—O11—C21	−92.0 (2)	C19—N3—C15—C20	−176.2 (2)
N1—Dy2—O11—C21	150.4 (2)	Dy2—N3—C15—C20	2.1 (3)
N3—Dy2—O11—C21	13.0 (2)	C12—N2—C8—C9	0.0 (4)
O5—Dy2—N2—C8	−1.01 (17)	Dy2—N2—C8—C9	177.92 (19)
O1—Dy2—N2—C8	33.4 (2)	C12—N2—C8—C13	−180.0 (2)
O7—Dy2—N2—C8	−179.4 (2)	Dy2—N2—C8—C13	−2.1 (3)
O3—Dy2—N2—C8	−95.45 (18)	O6—C13—C8—N2	−173.9 (2)
O11—Dy2—N2—C8	−148.54 (16)	O5—C13—C8—N2	6.2 (3)
O9—Dy2—N2—C8	83.44 (18)	O6—C13—C8—C9	6.1 (4)
N1—Dy2—N2—C8	−51.34 (19)	O5—C13—C8—C9	−173.8 (3)
N3—Dy2—N2—C8	131.40 (17)	Dy2—O11—C21—O12	161.7 (3)
O5—Dy2—N2—C12	176.9 (2)	Dy2—O11—C21—C19	−17.3 (4)
O1—Dy2—N2—C12	−148.70 (17)	C8—N2—C12—C11	−0.7 (4)
O7—Dy2—N2—C12	−1.49 (17)	Dy2—N2—C12—C11	−178.6 (2)
O3—Dy2—N2—C12	82.45 (18)	C8—N2—C12—C14	179.8 (2)
O11—Dy2—N2—C12	29.4 (2)	Dy2—N2—C12—C14	1.9 (3)
O9—Dy2—N2—C12	−98.66 (19)	C10—C11—C12—N2	0.7 (4)
N1—Dy2—N2—C12	126.56 (18)	C10—C11—C12—C14	−179.9 (3)
N3—Dy2—N2—C12	−50.7 (2)	O8—C14—C12—N2	179.4 (2)
O5—Dy2—O3—C7	71.8 (2)	O7—C14—C12—N2	−1.0 (3)
O1—Dy2—O3—C7	−0.7 (2)	O8—C14—C12—C11	−0.1 (4)
O7—Dy2—O3—C7	−156.8 (2)	O7—C14—C12—C11	179.5 (3)
O11—Dy2—O3—C7	−79.3 (2)	Dy2—O9—C20—O10	174.2 (2)
O9—Dy2—O3—C7	134.5 (2)	Dy2—O9—C20—C15	−6.4 (3)
N2—Dy2—O3—C7	136.5 (2)	N3—C15—C20—O10	−178.0 (2)
N1—Dy2—O3—C7	−2.17 (19)	C16—C15—C20—O10	3.3 (4)
N3—Dy2—O3—C7	−110.7 (2)	N3—C15—C20—O9	2.5 (3)
O5—Dy2—O9—C20	−154.7 (2)	C16—C15—C20—O9	−176.1 (3)
O1—Dy2—O9—C20	−73.8 (2)	Dy2—O3—C7—O4	−173.2 (2)
O7—Dy2—O9—C20	75.2 (2)	Dy2—O3—C7—C5	5.0 (3)
O3—Dy2—O9—C20	140.5 (2)	N1—C5—C7—O4	172.6 (2)
O11—Dy2—O9—C20	4.1 (2)	C4—C5—C7—O4	−7.1 (4)
N2—Dy2—O9—C20	138.6 (2)	N1—C5—C7—O3	−5.8 (3)
N1—Dy2—O9—C20	−102.5 (2)	C4—C5—C7—O3	174.5 (3)
N3—Dy2—O9—C20	5.4 (2)	N1—C1—C2—C3	−1.1 (4)
O5—Dy2—O7—C14	−1.0 (2)	C6—C1—C2—C3	179.7 (3)
O1—Dy2—O7—C14	139.05 (19)	N2—C8—C9—C10	0.7 (4)
O3—Dy2—O7—C14	−75.7 (2)	C13—C8—C9—C10	−179.3 (3)
O11—Dy2—O7—C14	−156.8 (2)	N1—C5—C4—C3	−3.3 (4)
O9—Dy2—O7—C14	73.8 (2)	C7—C5—C4—C3	176.3 (3)
N2—Dy2—O7—C14	0.9 (2)	C15—N3—C19—C18	−0.6 (4)
N1—Dy2—O7—C14	−108.4 (2)	Dy2—N3—C19—C18	−178.9 (2)
N3—Dy2—O7—C14	136.3 (2)	C15—N3—C19—C21	179.4 (2)
O5—Dy2—O1—C6	−76.9 (2)	Dy2—N3—C19—C21	1.2 (3)
O7—Dy2—O1—C6	133.71 (19)	O12—C21—C19—N3	−169.3 (3)
O3—Dy2—O1—C6	−0.8 (2)	O11—C21—C19—N3	9.8 (4)
O11—Dy2—O1—C6	73.1 (2)	O12—C21—C19—C18	10.8 (5)
O9—Dy2—O1—C6	−157.6 (2)	O11—C21—C19—C18	−170.2 (3)
N2—Dy2—O1—C6	−108.3 (2)	N3—C15—C16—C17	−2.3 (4)
N1—Dy2—O1—C6	0.76 (18)	C20—C15—C16—C17	176.2 (3)
N3—Dy2—O1—C6	136.4 (2)	C15—C16—C17—C18	0.4 (5)
O1—Dy2—O5—C13	−151.7 (2)	C16—C17—C18—C19	1.4 (5)
O7—Dy2—O5—C13	7.1 (3)	N3—C19—C18—C17	−1.3 (4)
O3—Dy2—O5—C13	77.6 (2)	C21—C19—C18—C17	178.7 (3)
O11—Dy2—O5—C13	139.3 (2)	C12—C11—C10—C9	0.1 (4)
O9—Dy2—O5—C13	−72.8 (2)	C8—C9—C10—C11	−0.8 (4)
N2—Dy2—O5—C13	5.2 (2)	C1—C2—C3—C4	0.4 (5)
N1—Dy2—O5—C13	141.9 (2)	C5—C4—C3—C2	1.7 (4)
N3—Dy2—O5—C13	−99.8 (2)	N8—C30—C29—N9	0.2 (4)
O5—Dy2—N3—C19	−155.57 (17)	C30—C29—N9—C28	0.0 (5)
O1—Dy2—N3—C19	−102.47 (19)	C29—N9—C28—N8	−0.2 (5)
O7—Dy2—N3—C19	75.94 (19)	N9—C28—N8—C30	0.4 (4)
O3—Dy2—N3—C19	28.1 (2)	C29—C30—N8—C28	−0.4 (4)
O11—Dy2—N3—C19	−6.39 (18)	C26—N7—C25—N6	−0.2 (4)
O9—Dy2—N3—C19	174.7 (2)	C25—N7—C26—C27	0.1 (4)
N2—Dy2—N3—C19	121.87 (18)	N7—C25—N6—C27	0.3 (4)
N1—Dy2—N3—C19	−55.4 (2)	N7—C26—C27—N6	0.1 (4)
O5—Dy2—N3—C15	26.2 (2)	C25—N6—C27—C26	−0.2 (4)
O1—Dy2—N3—C15	79.27 (18)	N5—C23—C24—N4	−1.1 (4)
O7—Dy2—N3—C15	−102.32 (19)	C24—C23—N5—C22	1.0 (4)
O3—Dy2—N3—C15	−150.11 (17)	N4—C22—N5—C23	−0.6 (4)
O11—Dy2—N3—C15	175.4 (2)	N5—C22—N4—C24	−0.1 (4)
O9—Dy2—N3—C15	−3.53 (17)	C23—C24—N4—C22	0.8 (4)
N2—Dy2—N3—C15	−56.4 (2)		

### Hydrogen-bond geometry (Å, °)

**Table:**

*D*—H···*A*	*D*—H	H···*A*	*D*···*A*	*D*—H···*A*
N4—H4A···O14^i^	0.94 (2)	2.50 (3)	3.286 (4)	141 (4)
N5—H5···O12^i^	0.86	2.27	3.121 (5)	171
N8—H8A···O10^ii^	0.96 (2)	2.29 (2)	3.241 (4)	172 (4)
N7—H7A···O10^iii^	0.87 (2)	1.82 (2)	2.695 (4)	177 (4)
N7—H7A···O9^iii^	0.87 (2)	2.60 (3)	3.127 (3)	120 (3)
N6—H6···O14^iv^	0.86	1.89	2.731 (4)	167
O13—H2W···O15^iv^	0.85	2.02	2.828 (4)	159
O13—H1W···O11	0.85	2.05	2.896 (3)	174
O13—H1W···O12	0.85	2.61	3.192 (4)	127
O14—H3W···O4	0.85	1.91	2.756 (3)	178
O14—H4W···O6^v^	0.85	2.06	2.842 (3)	153
O15—H5W···O2^vi^	0.85	2.22	3.061 (4)	169
O15—H6W···O4	0.85	2.00	2.845 (4)	172

Symmetry codes: (i) *x*+1, *y*, *z*; (ii) *x*−1, *y*, *z*+1; (iii) *x*−1, *y*+1, *z*; (iv) −*x*, −*y*+1, −*z*+1; (v) −*x*+1, −*y*, −*z*+1; (vi) −*x*+1, −*y*+1, −*z*+1.
